# Vascularity of the urethra in continent women using colour doppler high-frequency endovaginal ultrasonography

**DOI:** 10.1186/2193-1801-3-619

**Published:** 2014-10-20

**Authors:** Farah Lone, Abdul H Sultan, Aleksandra Stankiewicz, Ranee Thakar, Andrzej Pawel Wieczorek

**Affiliations:** Subspecialty trainee Urogynaecology, Department of Obstetrics and Gynaecology, Croydon University Hospital, Croydon, UK; Department of Obstetrics and Gynaecology, Croydon University Hospital, Croydon, UK; Clinical Research Fellow, Department of Obstetrics and Gynaecology, Croydon University Hospital, Croydon, UK; Consultant Obstetrician and Urogynaecologist, Department of Obstetrics and Gynaecology, Croydon University Hospital, Croydon, UK; Consultant Radiologist, Department of Diagnostic Imaging, Children’s Teaching Hospital, Lublin Medical University, Lublin, Poland

**Keywords:** Colour doppler, Endovaginal ultrasound, Urethra, Vascularity, Pixel flux, Urinary incontinence

## Abstract

**Objectives:**

To assess the urethral vascularity in continent women using colour doppler high frequency endovaginal ultrasonography (EVUS).

**Methods:**

We recruited 61 continent women attending gynaecology clinics between July and October 2009. Exclusion criteria included symptoms of urinary incontinence, voiding dysfunction, pelvic organ prolapse or urinary tract infection. The participants underwent EVUS using high frequency (9-12 MHz) biplane transducer (type 8848 BK Medical), according to a standardised protocol. Colour Doppler US was performed in sagittal plane and in transverse plane at the level of the mid-urethra. Ten seconds video files were recorded and following vascular parameters: flow velocity (V_mix_), area of the vessels (A_mix_), intensity of vascularity (I_mix_), pulsatility index (PI_mix_) and resistance index (RI_mix)_ was evaluated.

**Results:**

There were 30 nulliparous (49.2%) women and 31 multiparous women (50.8%) with a mean (±SD) age of 32 (±4) and 46 (±6) years respectively. Significant impairment of vascularity was observed in multiparous patients as compared to nulliparous and was reflected by increased values of RI_mix_ (p < 0.001) and PI_mix_ (p < 0.001), and decreased values of V_mix_ (p < 0.001), A_mix_ (p < 0.001), I_mix_ (p < 0.001) in axial and midsagittal sections of the midurethra. A significant decrease of mean value ± SD of I_mix_- from 0.02 ± 0.02 in nulliparous to 0.005 ± 0.01 in multiparous was observed.

Cronbach alpha, used to assess vascular correlations and parity demonstrated a reduction when expressed only for vascular parameters, indicating that number of deliveries is an important factor while assessing urethral vascularity.

**Conclusions:**

Compared to continent nulliparous women, continent multiparous women demonstrated a significant reduction in the vascularity parameters in all measured variables when parity was accounted for.

**Advances in knowledge:**

This study provides the basis for further research in assessing urethral vascularity in women.

## Introduction

Vascularity within the urethral sub mucosa contributes to the normal tension of the urethral mucosal wall (Ashton-Miller & DeLancey 2007). Although transperineal ultrasound (TPUS) has previously been used in the assessment of urethral vascularity,(Dietz et al. 1999; Dietz & Clarke 2001) the examination is limited, by the use of low-frequency transducers (4–7 MHz), as these do not provide accurate visualisation of such a tiny vessels like those of urethra. Moreover, during TPUS, some artefacts could be produced due to excessive pressure on the urethra.

Colour Doppler high-frequency endovaginal ultrasound (EVUS) has been reported as a reliable tool in the assessment of urethral vascularity (Wieczorek et al. 2009; Wieczorek et al. 2012; Wieczorek et al. 2011) in continent nulliparous due to small array of the transducer, high frequency (9-12 MHz) and short distance between transducer and the examining organs providing high resolution which results in better visualization of the urethra. However, there are no studies assessing the vascular parameters in continent multiparous women. The Pixel Flux software used in this study is a reliable method of assessing vascularity of various organs, including urethra (Scholbach et al. 2006; Scholbach et al. 2004a; Scholbach et al. 2004b; Scholbach et al. 2005).

The aim of our study was to compare the vascular parameters in the midurethra in continent nulliparous and multiparous females using high-frequency 2D-EVUS in the colour doppler mode.

## Materials and methods

In this observational study, we recruited 61 continent women attending gynaecology clinics between July and October 2009. Thirty women in nulliparous group and 31 in multiparous group were included. These women had non-urogynaecological presenting symptoms like menorrhagia, dyspareunia, irregular vaginal bleeding, pelvic pain, vulvo-vaginal cysts, fibroids etc Exclusion criteria for the asymptomatic females included symptoms of urinary incontinence, voiding dysfunction, pelvic organ prolapse or urinary tract infections. A detailed history was taken and demographic data was collected which included age, parity and use of hormone replacement therapy.

All patients underwent vaginal examination performed by experienced clinicians (AHS, RT). As it is our routine practice to perform a vaginal examination as part of assessment of gynaecological symptoms, pelvic organ prolapse was ruled out.

The endovaginal ultrasound (EVUS) scan was performed by an investigator (FL) experienced in performing EVUS with the use of biplane electronic, high frequency (9-12 MHz) transducer (type 8848 BK-Medical, Herlev, Denmark)and Profocus ultrasound scanner (BK-Medical, Herlev, Denmark).

This transducer provides two-dimensional (2D) sagittal (linear array) and axial (transverse array) section of the anterior compartment focusing on the urethra in colour Doppler mode.

For ultrasound assessment, patients were asked to have a comfortably full bladder and no rectal or vaginal contrast was used. The examination was performed with the patient in the supine position.

**Figure 1:**
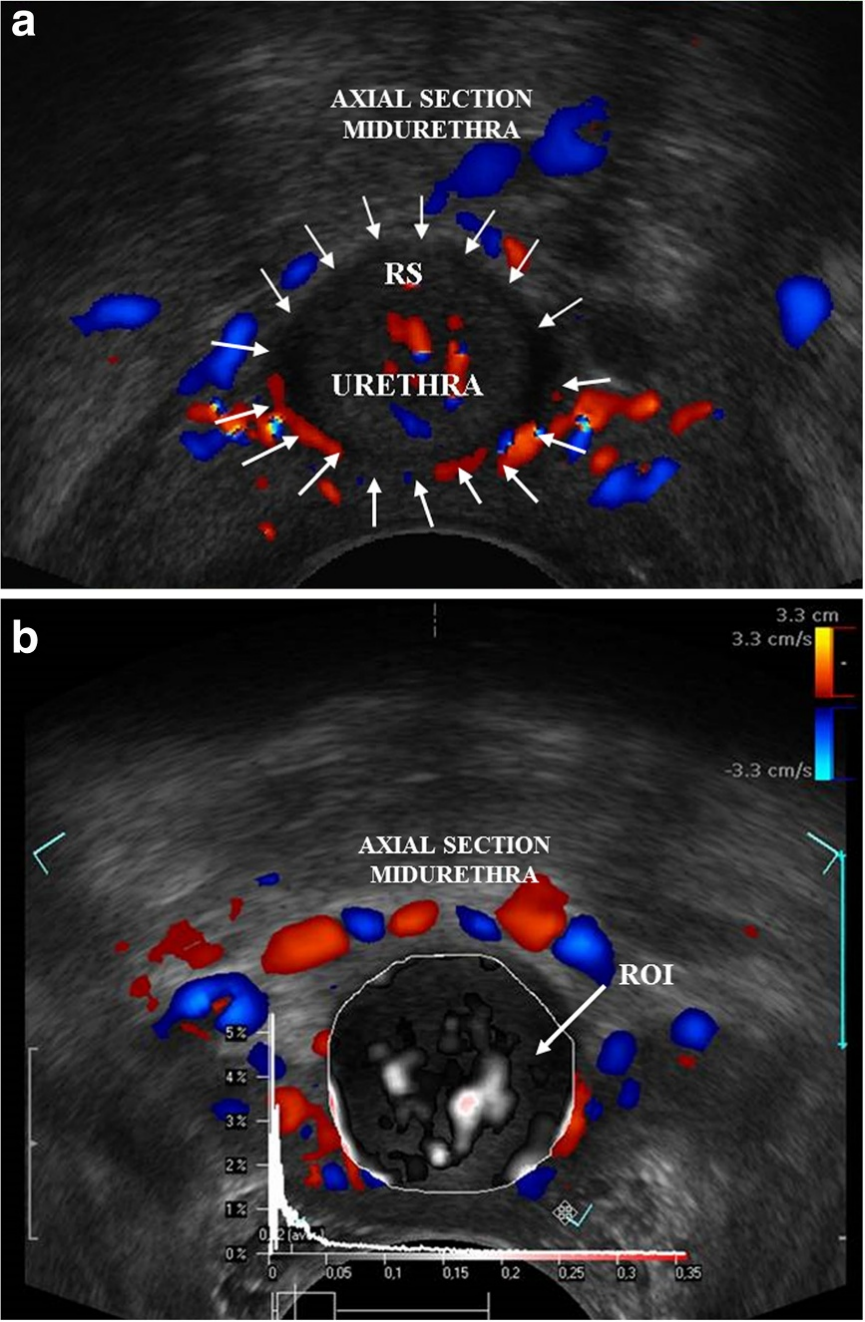
**Endovaginal US and Pixel Flux technique in axial section of the mid urethra. a** Endovaginal US with the use of high-frequency 180 degrees rotational transducer (type 8848, B-K Medical) in axial section of the mid urethra, colour Doppler mode. Arrows pointing the borders of the urethra. RS- rhabdosphincter. **b** Pixel Flux technique: Region of interest (ROI) in axial section of mid urethra showing the local perfusion relief.

**Figure 2:**
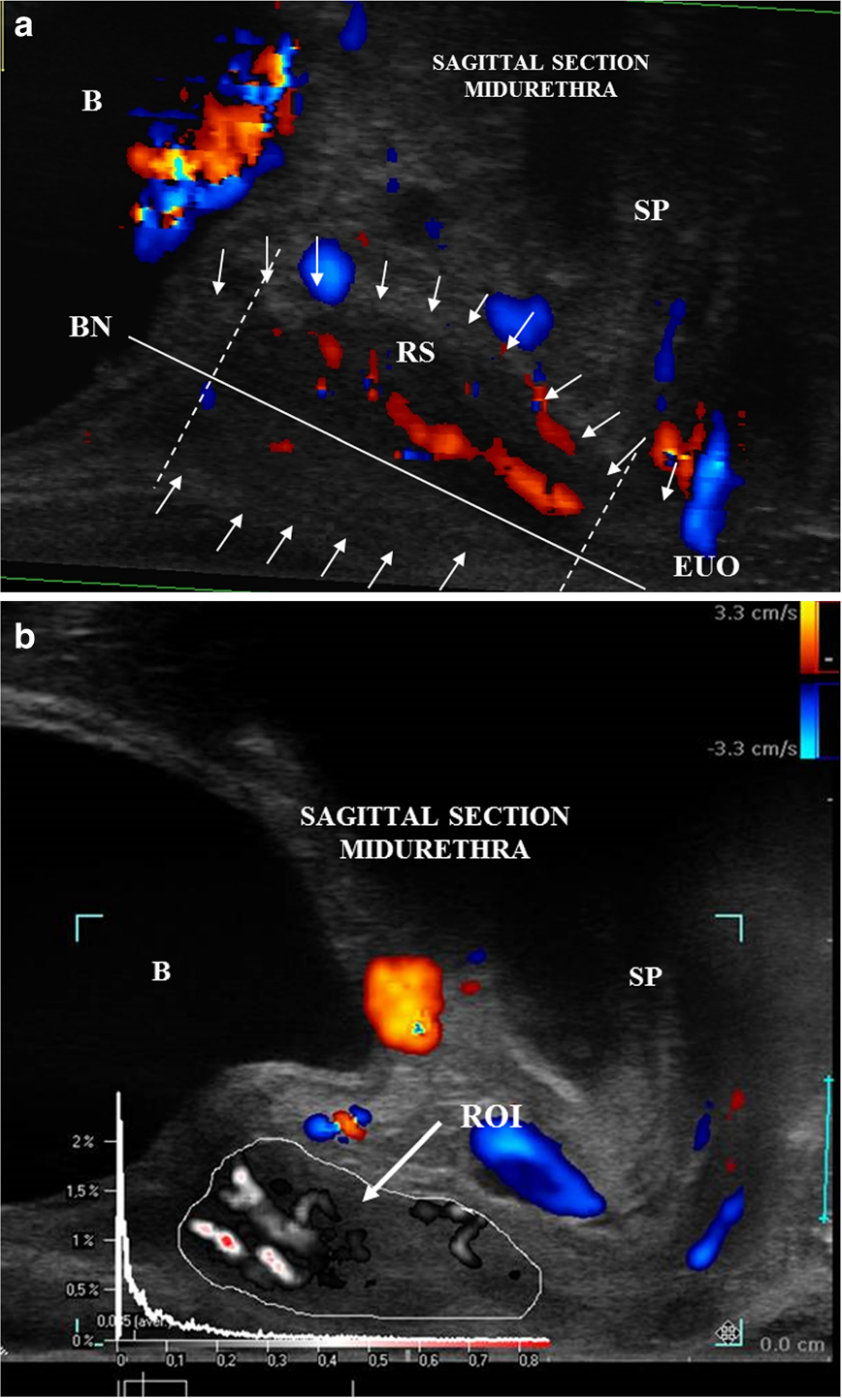
**Endovaginal US and Pixel Flux technique in midsagittal section of the mid urethra. a** Endovaginal US with the use of high-frequency 180 degrees rotational transducer (type 8848, B-K Medical) in midsagittal section of the urethra, colour Doppler mode. Arrows pointing the borders of the urethra. Area between the dotted lines demonstrates mid urethra. Straight line passing from bladder neck (BN) to external orifice of urethra (EUO) indicates urethral lumen. B- bladder, RS- rhabdosphincter, SP- symphysis pubis. **b** Pixel Flux technique: Region of interest (ROI) in midsagittal section of the urethra showing the local perfusion relief. B-bladder, SP- symphysis pubis.

The transducer was placed in the vagina in the neutral position to avoid any pressure on the surrounding structures distorting the anatomy (Santoro et al. 2009). The examination was performed at rest, with the use of colour doppler mode for the assessment of the urethral vascularity pattern, both in sagittal and axial sections of the urethra. This was recorded as video file (10 s or 3 heart cycles). Further off-line analysis with the use of Pixel Flux software was performed by two independent and experienced clinicians (AS and FL). The following vascular parameters were assessed: flow velocity (V_mix_), area of the vessels (A_mix_), intensity of vascularity (I_mix_), resistance index (RI_mix_) and pulsatility index (PI_mix_) in a predefined region of interest (ROI) in the axial [Figure 1a, 1b] and midsagittal [Figures 2a and 2b] sections. ROI was set on the external borders of the mid-urethra, comprising the lisosphincter and rhabdosphincter muscle [Figures 1b and 2b].

### Statistical analysis

Data analysis was carried out by SPSS version 13.

Descriptive analysis of frequency distribution was done. The differences between means of nulliparous and multiparous groups for all parameters were tested. The assumption of equal variances was tested by Levene's Test for equality of variances. If variables were homoscedastic, means were compared by *t*-test for independent sample calculated with pooled variances. For non homogenous variables the Behrens-Fisher T statistic was used and the Welch-Satterthwaite correction was made to the degrees of freedom. Statistically significant differences were assumed at p <0.05.

Cronbach alpha was used to assess item-total correlations between vascularity parameters including parity.

### Ethics approval

The study was approved by the National Research Ethics Service, Bromley Local Research Ethics Committee, South London REC office. (Ethics study number: 08/H0806/115). All women gave written informed consent.

## Results

Sixty one women comprising of 30 nulliparous and 31 multiparous women were included. The baseline characteristics of the study population are shown in Table 1. The mean (±SD) age was 32 (±4) years with an age range of 28-34 years in nulliparous group and 46 (±6) with an age range of (38-54) years in multiparous group. Median (range) of parity in multiparous group was 2 (0-6). Forty three women were British or of other white background origin, 12 women were Afro-Caribbean and 3 women were Asian in origin. The distribution was fairly similar in nulliparous as well as in multiparous group. In multiparous group, 6 women had previously undergone caesarean sections (3 women underwent 1 caesarean section each, and other 3 women had 2, 3 and 4 caesarean sections respectively). Of the 6 women who had undergone caesarean deliveries, five women had delivered by vaginal route as well.

**Table 1:** **Baseline characteristics of the study population (n = 61)**

	Nulliparous N = 30	Multiparous N = 31
Age (years)^a^	32 ± 4	46 ± 6
BMI (kg/m^2^)^a^	29.3 ± 6.5	30.1 ± 4.2
Parity^b^ 0	30 (100)	
1		9 (29)
2		9 (29)
3		5 (16)
4		4 (13)
5		3 (10)
6		1 (3)

^a^Data presented as mean ± SD.

^b^Number of patients (percentage).

One woman in the nulliparous group had premature ovarian failure and 2 women in multiparous group were postmenopausal and were using hormone replacement therapy.

**Figure 3:**
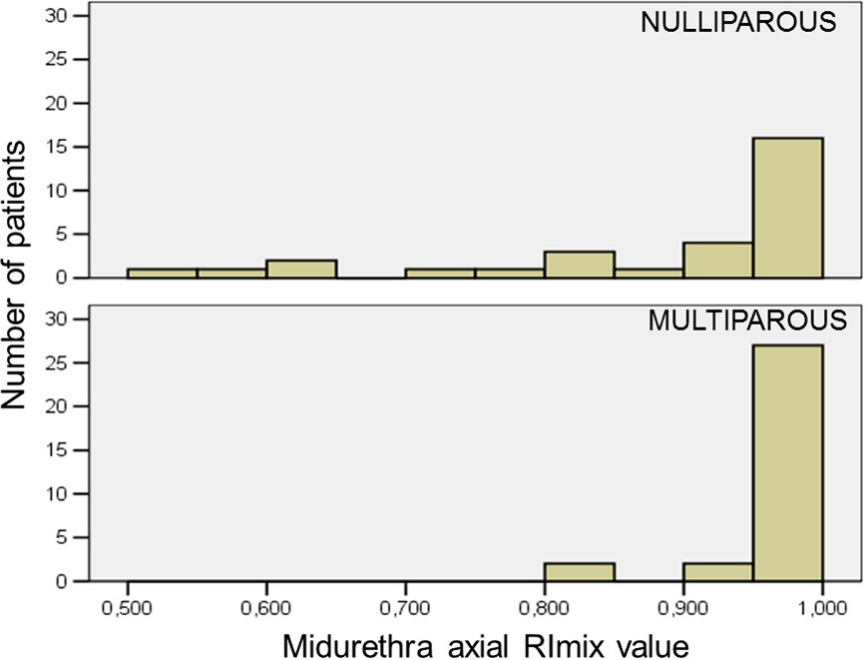
**The graphs presenting differences in values of RI**_**mix**_ **between nulliparous and multiparous patients measured in the axial section of the midurethra.** Majority of multiparous patients showed the value of RI _mix_ equal 1, while in nulliparous its values were more diverse, ranging from 0.5-1.

**Figure 4:**
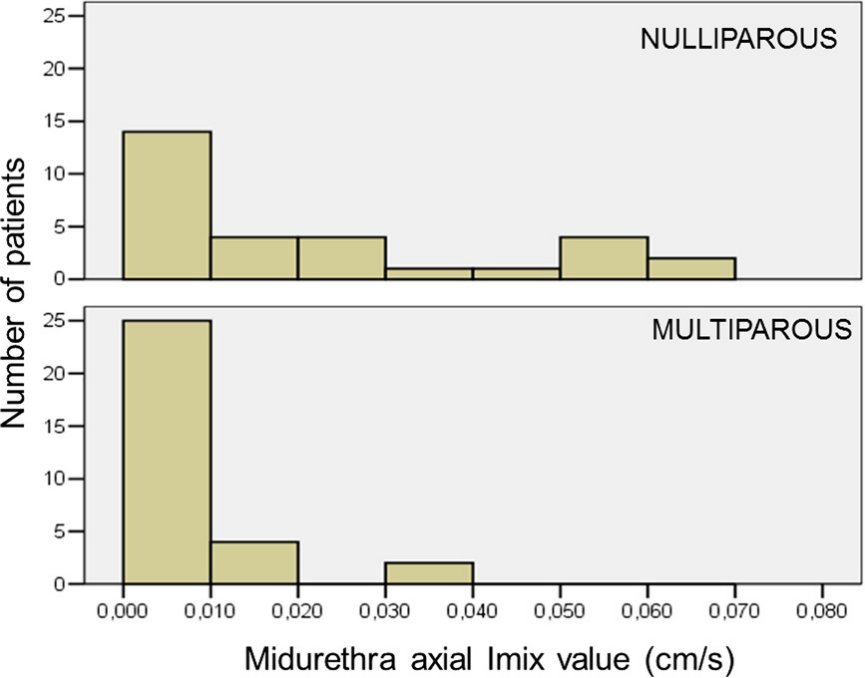
**The graphs presenting differences in values of the intensity of perfusion (I**_**mix**_ **) in the midurethra between nulliparous and multiparous patients, measured in the axial section of the urethra.** Majority of multiparous patients had the value of I_mix_ ≤0.01, and the highest value of I_mix_ = 0.04. In nulliparous patients the values of I_mix_ varied, ranging from ≤0.01- 0.07.

A comparison between nulliparous and multiparous was made and statistically significant differences were assumed at p <0.05. A significant reduction of vascularity was seen in axial and midsagittal sections of the midurethra of multiparous women as reflected by increased values of RI_mix_ [Figure 3] and PI_mix_ (p < 0.005 and p < 0.001 respectively) and decreased values of V_mix_, A_mix_, I_mix_ (Table 2). Impaired vascularity is demonstrated by the significant reduction of the mean value (SD) of I_mix_ from 0.02 (0.02) in nulliparous to 0.005 (0.01) in multiparous women [Figure 4].

**Table 2:** **Mean values and standard deviations (SD) of the vascular parameters obtained in the midurethra in axial and midsagittal planes**

PARAMETER	NULLIPAROUS mean (SD)	MULTIPAROUS mean (SD)	p-value
Midurethra axial V_mix_ (cm/s)	0.37(0.16)	0.18 (0.09)	<0.001
Midurethra axial A_mix_	0.05 (0.05)	0.02 (0.02)	<0.001
Midurethra axial I_mix_	0.02 (0.02)	0.005 (0.01)	<0.001
Midurethra axial RI_mix_	0.89 (0.15)	0.98 (0.05)	<0.005
Midurethra axial PI_mix_	2.34 (1.18)	3.18 (1.47)	0.01
Midurethra sagittal V_mix_ (cm/s)	0.41 (0.1)	0.18 (0.08)	<0.001
Midurethra sagittal A_mix_	0.1 (0.06)	0.02 (0.02)	<0.001
Midurethra sagittal I_mix_	0.01 (0.01)	0.01 (0.01)	0.01
Midurethra sagittal RI_mix_	0.62 (0.24)	0.99 (0.04)	<0.001
Midurethra sagittal PI_mix_	1.04 (0.62)	2.86 (1.29)	<0.001

Doppler analysis performed by two independent clinicians showed reproducible results for both axial and sagittal plane parameters.

Statistically significant differences were observed in the vascularity between nulliparous and multiparous women in all measured vascularity variables (Table 2). Item-total correlation (Cronbach’s alpha) between all vascular parameters including parity was 0.572 (p = 0000). Cronbach’s alpha decreased to 0.12 when expressed only for vascular parameters, indicating that number of deliveries is an important factor while assessing urethral vascularity.

## Discussion

We have found that compared to continent nulliparous women, continent multiparous women have a significantly reduced mid-urethral vascularity in all measured variables. A Pubmed search has revealed that there is no other published study comparing urethral vascularity in continent nulliparous and multiparous women.

The vascularity within the urethral sub mucosa contributes to the normal tension of the urethral mucosal wall playing a prominent role in the function of urethral complex and maintaining continence (Ashton-Miller & DeLancey 2007). Despite its importance, the assessment of vascular parameters describing urethral blood perfusion has not been comprehensively investigated. We analysed the vascular parameters in the urethra in continent women with the use of high-frequency EVUS and Pixel Flux- dedicated software for the assessment of blood perfusion in various organs (Scholbach et al. 2006; Scholbach et al. 2004a; Scholbach et al. 2004b; Scholbach et al. 2005).

The urethra is an organ consisting of three parts along its length (Ashton-Miller & DeLancey 2007) namely, intramural, midurethral and distal part. Each of them plays a different, but very important role in the proper functioning of the urethra. The intramural part creates a connection between the urinary bladder and the urethra and due to its muscular tone, maintains a tonic closure of the bladder neck at rest (Resnick & Yalla 1992). The midurethra consists of two layers; the internal layer is created by the lisosphincter longitudinal and circular layers while the external layer is the rhabdosphincter muscle. The lisosphincter is responsible for the tension and opening of the bladder neck during micturition, while the rhabdosphincter maintains proper closure pressures to keep the urethra closed (Resnick & Yalla 1992). The distal part of the urethra is a connection between the perineal membrane and the superficial perineal muscles.

The values of vascular parameters in the different parts of the urethra have been measured using various techniques (Scholbach et al. 2006; Siracusano et al. 2001). In the pioneering study by Wieczorek et al, on quantitative assessment of urethral vascularity in nulliparous women, compared to the other parts of the urethra, the midurethra appeared to have the highest value of V_mix_ and the lowest value of A_mix._ (Wieczorek et al. 2011). This high value of blood flow velocity reflects the high requirement of blood in the largest section of the urethra. As the midurethra with its vascular plexus seems to play the most important role in urethral physiology (Ashton-Miller & DeLancey 2007) we decided to focus on the assessment of vascular parameters in the midurethra, in continent females and to identify any differences in urethral vascularity parameters between the two groups of continent women. The results of our study obtained in the axial and sagittal plane in nulliparous continent women are similar to those obtained by Wieczorek et al. (Wieczorek et al. 2011).

In our study, the correlations between vascular parameters and parity, has indicated that the number of deliveries is an important factor influencing urethral vascularity. However, some studies performed with the use of spectral doppler in TPUS assessment of the urethral vascularity in incontinent multiparous have shown contradictory results i.e. RI was not correlated with parity (Hall et al. 2006). In the study by Siracusano et al. (Siracusano et al. 2001) colour doppler ultrasound of the female urethra in normal fertile young women identified a significantly greater RI in the proximal urethra compared to the middle and distal urethra. Similar results were obtained in the studies by Yang et al. (Yang et al. 2006) and Wieczorek et al. (Wieczorek et al. 2011) However all authors emphasised that the differences in obtained RI values may result from general limitations of doppler flow analysis in the urethra. Although the usefulness of colour and spectral doppler analysis of urethral vascularity has been widely described in the literature, (Siracusano et al. 2001; Yang et al. 2006; Kobata et al. 2008) the spectrum of blood flow and RI values are different.

It is known, that the doppler flow spectrum may be impaired by a number of factors such as ageing, body mass index, menopause, sex hormones profile,(Yang et al. 2006; Kobata et al. 2008; Tsai et al. 2001; Jármy-Di Bella et al. 2000; Palmieri et al. 2007; Miodrag et al. 1988) movement and breathing artifacts as well as by the ultrasonographic access and equipment used (Wieczorek et al. 2011). Until now, the most widely used ultrasound technique has been the transperineal/translabial approach. The limitation of this technique is that it is performed with 4-7 MHz convex transducer that does not allow precise delineation of various anatomical parts of the urethra. From the physics of ultrasound we know that, higher frequency provides better resolution and low frequency gives better penetration of the ultrasound beam. Therefore, precise assessement of the vasculature of a relatively small organ such as a female urethra, could not be performed using low frequency transducers and transperineal approach.

Moreover, in spectral doppler studies, the quality of perfusion is calculated only at single points of selected vessels, and RI describes perfusion only at two extreme points of a cardiac cycle (peak systole and end of diastole) (Tublin et al. 2003; Terslev et al. 2003; Govind et al. 2008). There exist also other methods offering a reliable, practical and non-invasive method for the assessment of vascularity such as three-dimensional power doppler angiography **(**3D-PDA) and virtual organ computer-aided analysis (VOCAL™) described in the assessment of ovarian and endometrial vascularity (Raine-Fenning et al. 2003), cervical vascularity(Basgul et al. 2007) and recently also placental volume and vascularity (Jones et al. 2011). However, as all ultrasound methods 3D-PDA and VOCAL may be impaired by movement/breathing artefacts, lack of standard protocol and predefined machine settings (fixed presets). By contrast the recent advances in ultrasound diagnostics, such as high-frequency EVUS enriched with Pixel Flux software enabling automatic calculation of five different vascular parameters of the perfusion within selected tissues or parts of organs (Wieczorek et al. 2011; Scholbach et al. 2006; Scholbach et al. 2004a; Scholbach et al. 2004b; Scholbach et al. 2005) with excellent intraobserver reliability (Wieczorek et al. 2011) appeared more reliable tool in the assessment of the urethral vascularity.

We acknowledge that the number of patients in our study is small. There were only three women in our study who were using hormone replacement therapy and this small number is unlikely to have affected the results. Although it would have been ideal to perform doppler assessment on all women at same phase of the menstrual cycle to control for the possible effect of hormonal fluctuation, it was not practically feasible.

We are not aware of another study comparing urethral vascularity in continent nulliparous and multiparous women. Moreover we used the 2D-EVUS according to a standardised protocol and with its inherent less artefactual influences and limitations as compared to previously used TPUS.

## Conclusions

Continent multiparous women demonstrated a significant reduction in the vascularity parameters in the midurethra compared to continent nulliparous women. Therefore, parity must be taken into consideration in research studies involving Doppler assessment of the urethra.

This study has enabled identification of normal urethral vascular parameters in asymptomatic women. These baseline values can now be used for further evaluation of these parameters in incontinent female patients.
